# Anti-CCR4 treatment depletes regulatory T cells and leads to clinical activity in a canine model of advanced prostate cancer

**DOI:** 10.1136/jitc-2021-003731

**Published:** 2022-01-31

**Authors:** Shingo Maeda, Tomoki Motegi, Aki Iio, Kenjiro Kaji, Yuko Goto-Koshino, Shotaro Eto, Namiko Ikeda, Takayuki Nakagawa, Ryohei Nishimura, Tomohiro Yonezawa, Yasuyuki Momoi

**Affiliations:** 1 Department of Veterinary Clinical Pathobiology, Graduate School of Agricultural and Life Sciences, The University of Tokyo, Tokyo, Japan; 2 Veterinary Medical Center, The University of Tokyo, Tokyo, Japan; 3 Molecular Diagnostic Laboratory, Veterinary Medical Center, The University of Tokyo, Tokyo, Japan; 4 Laboratory of Veterinary Surgery, Graduate School of Agricultural and Life Sciences, The University of Tokyo, Tokyo, Japan

**Keywords:** prostatic neoplasms, translational medical research, immunotherapy, biomarkers, tumor

## Abstract

**Background:**

Targeting regulatory T cell (Treg) infiltration is an emerging strategy for cancer immunotherapy. However, its efficacy in advanced prostate cancer remains unclear. Here, we showed the therapeutic efficacy of anti-Treg treatment in a canine model of advanced prostate cancer.

**Methods:**

We used dogs with naturally occurring prostate cancer to study the molecular mechanism underlying Treg infiltration and the effect of anti-Treg treatment. Tumor-infiltrating Tregs was evaluated by immunohistochemistry, and the association with prognosis was examined in dogs with spontaneous prostate cancer. The molecular mechanism of Treg infiltration was explored by RNA sequencing and protein analyses. A non-randomized canine clinical trial was conducted to define the therapeutic potential of anti-Treg treatment for advanced prostate cancer. Human prostate cancer datasets were analyzed to compare gene expression in dogs and humans.

**Results:**

Tumor-infiltrating Tregs were associated with poor prognosis in dogs bearing spontaneous prostate cancer. RNA sequencing and protein analyses showed a possible link between the CCL17–CCR4 pathway and the increase of tumor-infiltrating Tregs. Dogs with advanced prostate cancer responded to mogamulizumab, a monoclonal antibody targeting CCR4, with decreased circulating Tregs, improved survival, and low incidence of clinically relevant adverse events. Urinary CCL17 concentration and BRAF^V595E^ mutation were independently predictive of the response to mogamulizumab. Analysis of a transcriptomic dataset of human prostate cancer showed that the CCL17–CCR4 axis correlated with Foxp3. In silico survival analyses revealed that high expression of CCL17 was associated with poor prognosis. Immunohistochemistry confirmed that tumor-infiltrating Tregs expressed CCR4 in human patients with prostate cancer.

**Conclusions:**

Anti-Treg treatment, through CCR4 blockade, may be a promising therapeutic approach for advanced prostate cancer in dogs and some population of human patients.

## Background

Prostate cancer is the most common malignancy in men, with an estimated 1.2 million new cancer cases and 359 000 deaths annually worldwide.^1^ Androgen deprivation therapy is a first-line treatment for advanced prostate cancer. This treatment alone, or in combination with chemotherapy, is initially effective in approximately 80%–90% of advanced prostate cancer cases. However, the disease eventually progresses to castration-resistant prostate cancer within months or years.^2^ Metastatic castration-resistant prostate cancer (mCRPC) has no curative treatment options and is associated with a poor prognosis. Although several treatments have been approved for mCRPC after progression with docetaxel chemotherapy, new treatment options that provide durable disease control are still needed.

Foxp3-expressing regulatory T cells (Tregs) play a role not only in the suppression of immune response against self-antigens but also in tumor progression by inhibiting the antitumor immunity. In humans, Treg infiltration has been observed in certain tumor tissues and has been associated with the progression of cancer and prognosis.^3^ High infiltration of Tregs into tumor tissues correlates with poor prognosis in patients with melanoma, hepatocellular carcinoma, lung carcinoma, ovarian cancer, breast cancer, pancreatic cancer, and prostate cancer.^3 4^ In tumor-bearing mice and humans with solid tumors, anti-Treg immunotherapy is under investigation.^5–10^ However, the role of Tregs and the therapeutic potential of its depletion in prostate cancer are unknown.

Although rodent models are indispensable in cancer research, the controlled environment under which highly inbred rodents are kept offers completely different settings from the diverse conditions prevalent in human cancer. Preclinical studies using murine models have often failed to predict the results of human clinical trial.^11^ The average rate of successful translation from rodent models to clinical cancer trials is less than 10%.^12^ In fact, clinical trials in patients with mCRPC using cytotoxic T-lymphocyte antigen 4 (CTLA-4) or programmed cell death protein 1 (PD-1)/PD-L1 inhibitors have been less satisfactory, with limited survival benefit when administered as a monotherapy in unselected patients than mice-based preclinical studies.^13–15^ Mouse models of prostate cancer are inadequate because of the lack of several features, such as genetic heterogeneity, molecular complexity, immune responses, symptoms, disease progression, metastatic behavior, and long-term evaluation. More relevant animal models of advanced prostate cancer are warranted to study the disease.

The canine prostate gland shares morphological and functional similarities with the human prostate. Dogs are the only animals to present with a significant incidence of spontaneous prostate cancer, with clinical features, including late age at onset and metastatic patterns resembling those in humans.^16 17^ Therefore, naturally occurring prostate cancer in companion dogs could serve as a bridge between laboratory animal models and human patients. Unlike humans, most dogs with prostate cancer present with an advanced and aggressive disease. Local invasion to the urethra, bladder trigone, and ureter is common and causes obstruction of urine outflow leading to hydronephrosis. Metastases have been reported in >40% of dogs at diagnosis and in approximately 80% at death, with spread primarily to the locoregional lymph nodes, lungs, liver, and bone.^18^ Dogs with prostate cancer often develop bone metastases in the lumbar vertebra, pelvis, and/or femur, associated with pain and neurological deficits.^18^ An important difference between human and canine prostate cancer is the role of androgen. Canine prostate cancer usually does not respond to androgen deprivation therapy or surgical castration.^19^ Since dogs have a shorter life span than humans, clinical trials using dogs can be conducted in a relatively shorter period. Thus, the canine prostate cancer model could be informative to study the pathogenesis of advanced prostate cancer, especially mCRPC, and to assess biomarkers and therapeutics.

Here, we show that Tregs in the tumor microenvironment are associated with poor prognosis in dogs with spontaneous prostate cancer, and that there is a link between Treg migration and C-C chemokine ligand 17 (CCL17)–CCR4 pathway. We also demonstrate the therapeutic efficacy of anti-CCR4 treatment in dogs with prostate cancer. Furthermore, we show that the CCL17–CCR4 axis is associated with Treg infiltration and poor prognosis in patients with human prostate cancer. The canine model of advanced prostate cancer paves the way for the translation of the anti-Treg immunotherapy to human patients with advanced prostate cancer.

## Methods

### Canine prostate cancer model and sample collection

Characteristics of dogs used for histological, mRNA expression, and protein analyses are presented in online supplemental table S1. For histological analysis, archival formalin-fixed, paraffin-embedded prostate cancer tissues were obtained from 18 dogs at the Veterinary Medical Center of the University of Tokyo (VMC-UT). All dogs underwent radical cystoprostatectomy. The diagnosis of prostate cancer was confirmed by histopathology. Tumor stage was defined as per the WHO criteria for canine prostate cancer.^20^ Normal canine prostate tissues were obtained from nine healthy beagles euthanized for another experimental purpose. Survival time and current status (alive, deceased, or lost) of all dogs were determined by medical record or interview. Overall survival (OS) in dogs that underwent radical cystoprostatectomy was defined as the time from surgery to the established cause of death of the animal at the end of the study (April 9, 2019).

10.1136/jitc-2021-003731.supp1 Supplementary data

Fresh tumor tissues from 18 dogs with prostate cancer were analyzed for mRNA expression (online supplemental table S1). Snap-frozen prostate tissues collected from five healthy beagles were used as normal control. For ELISA, serum samples were collected from 19 dogs with prostate cancer and 10 healthy dogs (online supplemental table S1). Fresh urine samples were collected using a urethral catheter from 19 dogs with prostate cancer and 14 healthy dogs (online supplemental table S1).

### Human samples

We obtained paraffin-embedded sections of prostate specimens from 6 healthy volunteers and 11 patients with prostate cancer (OriGene Technologies). Characteristics of human patients with prostate cancer are summarized in online supplemental table S2.

10.1136/jitc-2021-003731.supp2 Supplementary data

### Immunohistochemistry

The expression of Foxp3 and CCR4 was examined by immunohistochemistry and immunofluorescence with 4 μm-thick paraffin-embedded sections.^21^ Detailed information is provided in online supplemental materials.

10.1136/jitc-2021-003731.supp3 Supplementary data

### Canine prostate cancer RNA sequencing (RNA-Seq)

Total RNA was extracted from prostate cancer and normal prostate tissues using the RNeasy Mini Kit (Qiagen). RNA integrity was examined with an Agilent 2100 Bioanalyzer (Agilent Technologies) and RNA integrity number values of all samples were >7. Sequencing libraries were prepared with the TruSeq Stranded mRNA Library Prep Kit for NeoPrep (Illumina). RNA-Seq (75 bp paired end) was conducted using NextSeq 500 (Illumina) with the High Output Kit (Illumina), and a minimum of 35 million read-pairs was generated for each sample.

Quality controls and adaptor trimmings of fastq files for each sample were performed using the Trim Galore software based on FastQC and Cutadapt V.0.6.3 (https://www.bioinformatics.babraham.ac.uk/projects/trim_galore/). Trimmed fastq data were mapped to canine genomes (CanFam3.1) by STAR V.2.7.3a,^22^ and transcript abundance was estimated using RSEM V.1.3.3^23^ with gene transfer file for Ensembl (CanFam3.1.98, https://www.ensembl.org). These gene count data were used to normalize and extract differential gene expressions with an EdgeR-based R package, TCC V.1.26.0.^24^ The results of normalized expression gene data between two groups were scaled to have a mean=0 and SD=1 (z-score) by R package, genefilter V.1.68.0, and visualized using a volcano plot by R V.3.6.1.

The datasets used and/or analyzed during the current study are available from the corresponding author on reasonable request and will also be available at the DDBJ Sequenced Read Archive repository (https://www.ddbj.nig.ac.jp/index-e.html) with accession number DRA011773.

### Bioinformatic analysis of human prostate cancer

Datasets for human metastatic or nonmetastatic prostate cancer were accessed and BRAF gene alterations were analyzed through cBioPortal.^25–32^ A normalized mRNA expression dataset for human prostate cancer (The Cancer Genome Atlas (TCGA), PanCancer Atlas) was accessed and downloaded from the cBioPortal.^28^ Detailed information is provided in online supplemental materials.

### Quantitative real-time PCR

We quantified mRNA expression levels of interleukin (IL)-10, transforming growth factor beta (TGF-β), and chemokines identified in the RNA-Seq analysis using two-step real-time PCR (Thermal Cycler Dice Real Time System, Takara Bio). The ribosomal protein L13a (RPL13A) and RPL32 were used as reference genes. The primer pair sequences are shown in online supplemental table S3.

10.1136/jitc-2021-003731.supp4 Supplementary data

### ELISA

We measured canine CCL17 and CCL22 concentrations in the supernatants of serum and urine samples using the canine TARC/CCL17 and MDC/CCL22 ELISA kit (Cusabio), respectively. Urinary creatinine (Cre) concentration was measured using the LabAssay Creatinine kit (Wako), and urinary CCL17 and CCL22 concentrations were expressed as pg/mg of Cre.

### Flow cytometry

Blood samples were collected for isolation of peripheral blood mononuclear cells and analyzed for the expression of CD4, CD8, Foxp3, and CCR4 by flow cytometry. Detailed information is provided in online supplemental materials.

### Canine clinical trial design and interventions

The phase II prospective, non-randomized canine clinical trial was conducted at the VMC-UT. The client-owned dogs with prostate cancer were treated with anti-CCR4 therapy in combination with piroxicam, the standard drug for canine prostate cancer. Age-matched, sex-matched, and tumor stage-matched dogs with prostate cancer treated with piroxicam alone were used as a control arm for clinical response and survival. Dogs that had been administered chemotherapy or radiation therapy were excluded from this clinical trial. No placebo control, blinding, or randomization was performed in the study. Anti-CCR4 mAb (mogamulizumab, 1 mg/kg; Kyowa Hakko Kirin) was administered to dogs with metastatic or non-metastatic prostate cancer once every 3 weeks. The dosage and administration interval were based on a previous study.^21^ Treatment was continued until dogs experienced disease progression, had unacceptable toxicity, or the owner wished to discontinue the trial. Piroxicam (0.3 mg/kg, Pfizer) was administered every 24 hours in combination with mogamulizumab. Characteristics of dogs used in the clinical trial are summarized in online supplemental table S4.

10.1136/jitc-2021-003731.supp5 Supplementary data

### Clinical assessment

Dogs were evaluated for clinical responses and toxicity at least once every 3 weeks by owner observations, physical exam, complete blood counts, serum chemical profiles, three-view thoracic and two-view abdominal radiography, and abdominal ultrasonography. A single ultrasound operator (SM) measured prostate masses following a standardized protocol.^33^ For each dog, longitudinal and transverse views of the prostate were obtained, and three measurements (height, width, and longitudinal length) were recorded. According to the canine response evaluation criteria in solid tumors,^34^ we defined the tumor response as follows: complete remission (CR, no cancer detected), partial response (PR, ≥30% decrease in the sum of the longest diameters of target lesions from baseline and no new tumor lesions), progressive disease (PD, ≥20% increase in the sum of the longest diameters or the development of new tumor lesions), and stable disease (SD; not meeting the criteria for CR, PR, or PD). In this canine clinical trial, progression-free survival (PFS) was defined as the time from the start of treatment until PD or death at the end of the study (March 1, 2021), and OS as the time from the start of treatment until death of the animal at the end of the study. Adverse events were assessed and classified according to the Veterinary Cooperative Oncology Group criteria.^35^

### Biomarkers

For biomarker assessment, we collected fresh urine samples before treatments for CCL17 measurements and BRAF^V595E^ mutation. Urinary CCL17 was assessed by ELISA, as described previously. BRAF^V595E^ mutations were examined by digital PCR assay using genomic DNA isolated from urine sediments, as previously described.^36^

### Statistical analysis

All data in bar graphs are presented as mean±SEM. We used the JMP Pro V.15.0 (SAS Institute) for statistical analyses. The Mann-Whitney U test was used for comparison between two groups. The Kruskal-Wallis test, followed by the Dunn test, was used for multiple comparisons. Correlation between two variables was evaluated using the Spearman rank correlation coefficient. The Cochran-Armitage test for trend was used to evaluate clinical response and treatment or BRAF^V595E^ mutation. Survival curves were generated using the Kaplan-Meier method and compared using the log-rank test. Cox proportional hazard model was used for multivariate analyses of survival. Statistical significance was defined as a p value of <0.05.

## Results

### Tumor-infiltrating Tregs associate with adverse outcome in canine prostate cancer

In certain tumors of dogs as well as of humans, Treg infiltration is associated with poor prognosis. We evaluated the abundance of Tregs in the tumor microenvironment and the association of their density and prognosis in dogs with spontaneous prostate cancer. Tissue samples were obtained from 18 dogs with prostate cancer; all these dogs underwent radical cystoprostatectomy (online supplemental table S1). According to the WHO TNM classification for canine prostate cancer,^20^ 1/18 (6%) tumors were classified as T1 (intracapsular tumor, surrounded by normal gland), 2/18 (11%) as T2 (diffuse intracapsular tumor), 8/18 (44%) as T3 (tumor extending beyond the capsule), and 7/18 (39%) as T4 (tumor fixed, or invading neighboring structures). Nodal metastasis was detected in seven (39%) dogs. No distant metastasis (to the lungs or bones) was observed. After radical cystoprostatectomy, 5 dogs received no treatment; 10 received cyclooxygenase (COX) inhibitors (piroxicam or carprofen); and 2 were administered chemotherapy (cyclophosphamide or carboplatin) in combination with COX inhibitors.

**Figure 1:**
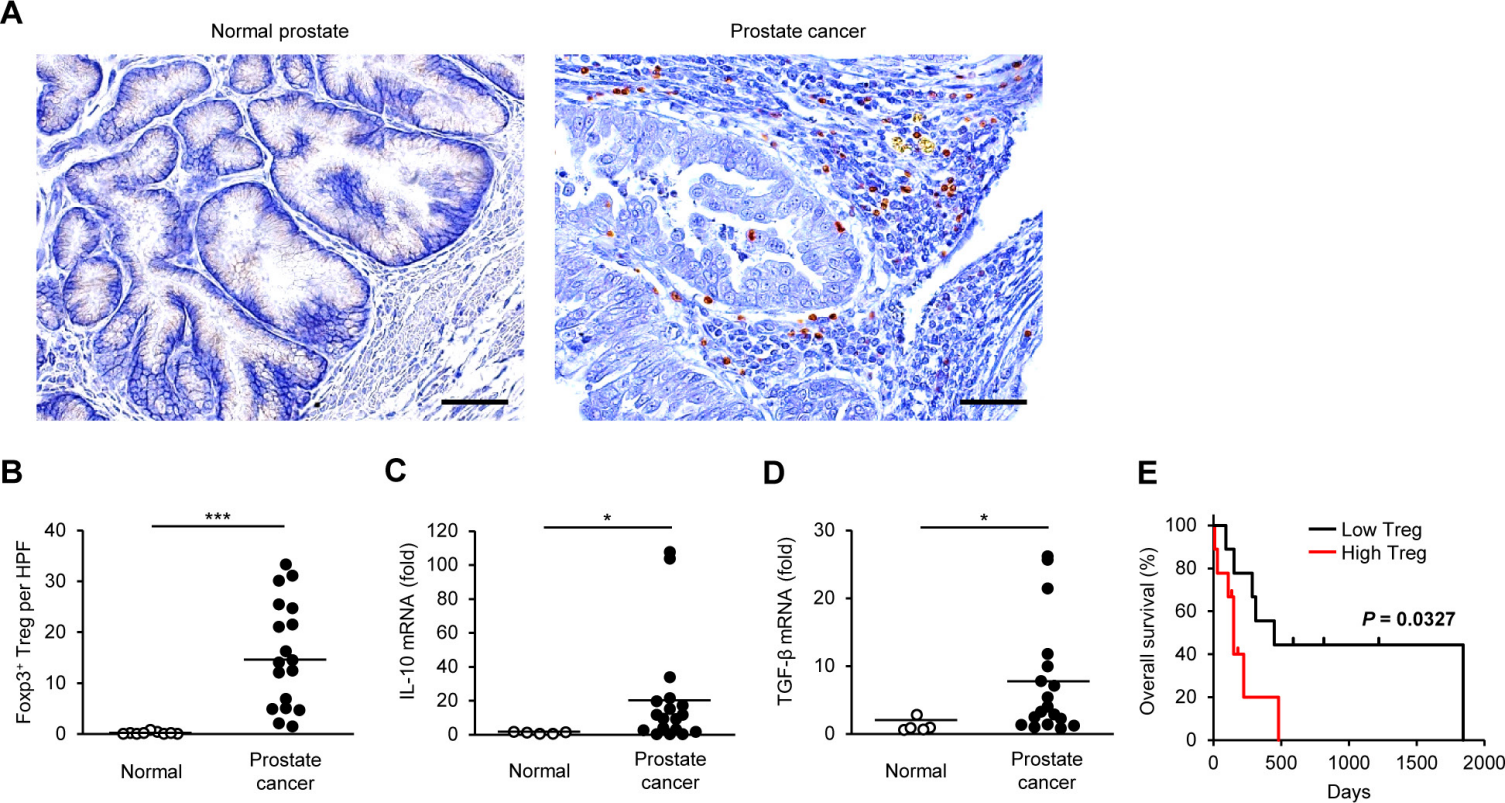
The number of tumor-infiltrating Tregs is associated with adverse outcome in dogs with prostate cancer. (A) Representative images of immunohistochemistry for Foxp3 in canine normal prostate and prostate cancer. Scale bar, 50 µm. (B) The number of Foxp3^+^ Tregs in the prostate of normal dogs (n=9) and dogs with prostate cancer (n=18). Median values are depicted by horizontal lines. (C, D) Expression of IL-10 (C) and TGF-β (D) mRNA in the prostate of normal dogs (n=5) and dogs with prostate cancer (n=18). Mean values are depicted by horizontal lines. (E) Kaplan-Meier curves of overall survival according to the number of intratumoral Tregs in dogs with prostate cancer (n=18). Cases were classified as having a high or low density of Foxp3^+^ Tregs according to the median number (n=9 each). Log-rank test. *P<0.05, ***P<0.001; non-parametric Mann-Whitney U test. IL, interleukin; TGF-β, transforming growth factor beta; Treg, regulatory T cell.

We visualized the expression of Foxp3 using immunohistochemistry and evaluated the localization and number of Tregs. Only a few Foxp3^+^ Tregs were detected in the normal canine prostate, whereas they were observed both within the tumor and in the surrounding stroma in canine prostate cancer (figure 1A). Compared with normal controls, Tregs were more frequently detected in prostate cancer tissues (figure 1B). Gene expression of the immunosuppressive cytokines, IL-10 and TGF-β, were increased in canine prostate cancer compared with that in normal tissues (figure 1C, D).

During the follow-up period, 13 of 18 dogs died (12 from progression of prostate cancer and 1 from disseminated intravascular coagulation). At the end of the study period, five dogs were alive. The median OS in dogs with prostate cancer was 201.5 days (range 8–1841). Based on the median number, we classified each prostate cancer case as having a high or low density of Foxp3^+^ Tregs. The OS for cases with high Tregs was shorter than that for cases with low Tregs (figure 1E). Further survival analysis showed that high IL-10 mRNA expression tended to be associated with adverse outcomes; however, neither IL-10 nor TGF-β expression was significantly associated with shorter OS (online supplemental figure S1). We performed Cox proportional hazard model to screen variables with prognostic value. Among the candidate covariates, age, castration, BRAF^V595E^ mutation, tumor burden, and Treg infiltration were included. The variable individually associated with a shortened OS was only Treg infiltration (HR 3.9, 95% CI 1.1 to 14.0; p=0.035). These results suggest that Tregs in the tumor microenvironment is associated with adverse outcome in dogs with prostate cancer.

10.1136/jitc-2021-003731.supp6 Supplementary data

### CCL17 expression is increased in canine prostate cancer

**Figure 2:**
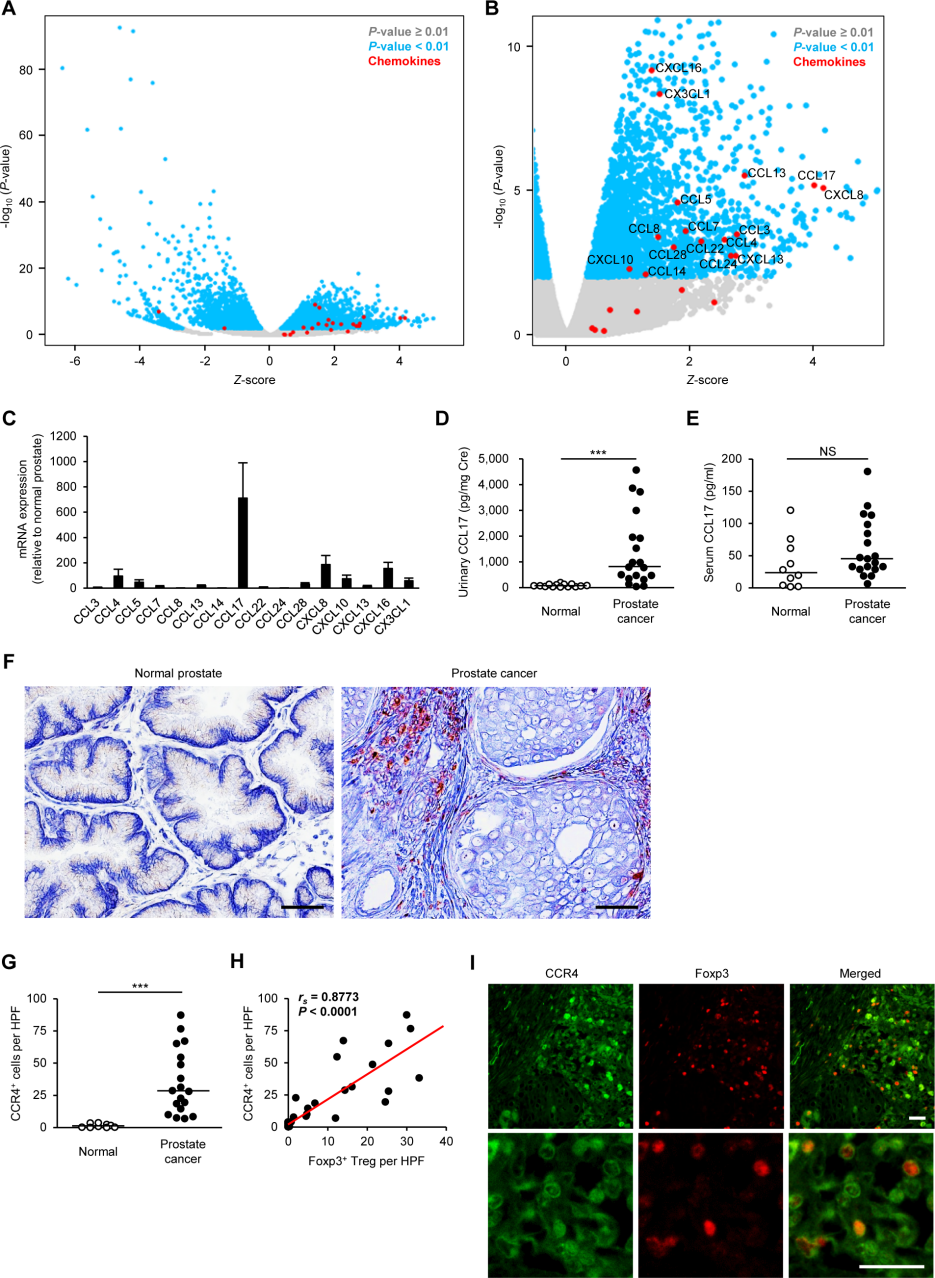
CCL17–CCR4 pathway is associated with the infiltration of Tregs into the tumor tissue in dogs with prostate cancer. (A) Volcano plot showing z-score for differentially expressed genes between canine normal prostate (n=4) and prostate cancer (n=14) as determined by RNA-Seq. (B) Volcano plot showing z-score for upregulated genes. Chemokine genes are enriched in upregulated genes. (C) Chemokine mRNA expression in canine prostate cancer (n=18) relative to that in the normal prostate (n=5) as determined by quantitative real-time PCR. (D) Urinary CCL17 concentration in normal dogs (n=14) and in dogs with prostate cancer (n=19). Mean values are depicted by horizontal lines. (E) Serum CCL17 concentration in normal dogs (n=10) and in dogs with prostate cancer (n=19). (F) Representative images of immunohistochemistry for CCR4 in canine normal prostate and prostate cancer. Scale bar, 50 µm. (G) The number of CCR4^+^ cells in the prostate of normal dogs (n=9) and dogs with prostate cancer (n=18). Median values are depicted by horizontal lines. (H) Correlation between Foxp3^+^ Tregs and CCR4^+^ cells in dogs with prostate cancer (n=18). Spearman rank correlation coefficient. (I) Representative images of immunofluorescence for CCR4 (green) and Foxp3 (red) in canine prostate cancer. Scale bar, 25 µm. NS, ***P<0.001; non-parametric Mann-Whitney U test. HPF, high-power field; NS, not significant; Treg, regulatory T cell.

To identify molecules inducing Treg infiltration in canine prostate cancer, we explored differentially expressed genes (DEGs) of chemokines for Tregs by RNA-Seq analysis. The analysis revealed several genes that were differentially regulated, with a p value <0.01, in canine prostate cancer when compared with normal controls. In total, 4599 DEGs showed significant changes between normal and prostate cancer tissues. Of these, 2301 DEGs were upregulated and 2298 were downregulated in canine prostate cancer (figure 2A). Sixteen chemokine genes were upregulated in canine prostate cancer compared with normal prostate (figure 2B). Quantitative real-time PCR showed approximately 700-fold increase in the expression of CCL17 gene in prostate cancer compared with the expression in normal canine prostate (figure 2C). Urinary CCL17 concentration was increased in dogs with prostate cancer (figure 2D). There was no significant difference in serum CCL17 concentration between normal dogs and dogs with prostate cancer (figure 2E). Compared with normal dogs, urinary and serum CCL22 tended to be increased in dogs with prostate cancer, but no significant difference was observed (online supplemental figure S2).

### Tumor-infiltrating Tregs express CCR4 in canine prostate cancer

CCL17 induces chemotaxis via the receptor CCR4.^37^ We examined CCR4^+^ cells in the tumor microenvironment of canine prostate cancer by immunohistochemistry. As expected, CCR4^+^ cells with a mononuclear lymphoid morphology were abundant in prostate cancer (figure 2F). More tumor-infiltrating CCR4^+^ cells were evident in dogs with prostate cancer (figure 2G), the density being positively correlated with the number of Foxp3^+^ Tregs (figure 2H). Double immunofluorescence analysis confirmed that Foxp3^+^ Tregs expressed CCR4 in the tumor microenvironment (figure 2I), indicating that CCL17–CCR4 axis possibly contributes to the infiltration of Tregs into canine prostate cancer tissues.

### BRAF^V595E^ mutation correlates with tumor-infiltrating Tregs and CCL17–CCR4 expression in canine prostate cancer

A somatic point mutation in the BRAF gene (BRAF^V595E^), which is homologous to the human BRAF^V600E^ mutation, is present in over 70% of dogs with bladder and prostate cancers.^38^ We have recently shown that BRAF^V595E^ mutation induces CCL17 production and contributes to Treg recruitment in dogs with bladder cancer.^36^ Thus, we investigated whether BRAF^V595E^ mutation influences tumor-infiltrating Tregs or the CCL17–CCR4 axis in dogs with prostate cancer. Of the 28 dogs with prostate cancer used in this study, BRAF^V595E^ mutation was detected in 21 (75%) cases (online supplemental table S1). Tumor-infiltrating Foxp3^+^ Tregs and CCR4^+^ cells were increased in cases with BRAF^V595E^ mutation in comparison to that in cases with wild-type BRAF (online supplemental figure S3). Moreover, urinary CCL17 concentration in cases with the BRAF^V595E^ mutation was higher than in cases with wild-type BRAF. The BRAF^V595E^ mutation was not detected in any normal dog (online supplemental table S1). Analysis of publicly available datasets of human prostate cancer showed that BRAF gene alterations (mutation, fusion, or copy number alteration) were found in 4.7%–6.5% and 3%–4% of patients with metastatic and non-metastatic prostate cancer, respectively (online supplemental figure S4).

### Anti-CCR4 treatment depletes Tregs and leads to clinical activity in spontaneous canine prostate cancer

**Figure 3:**
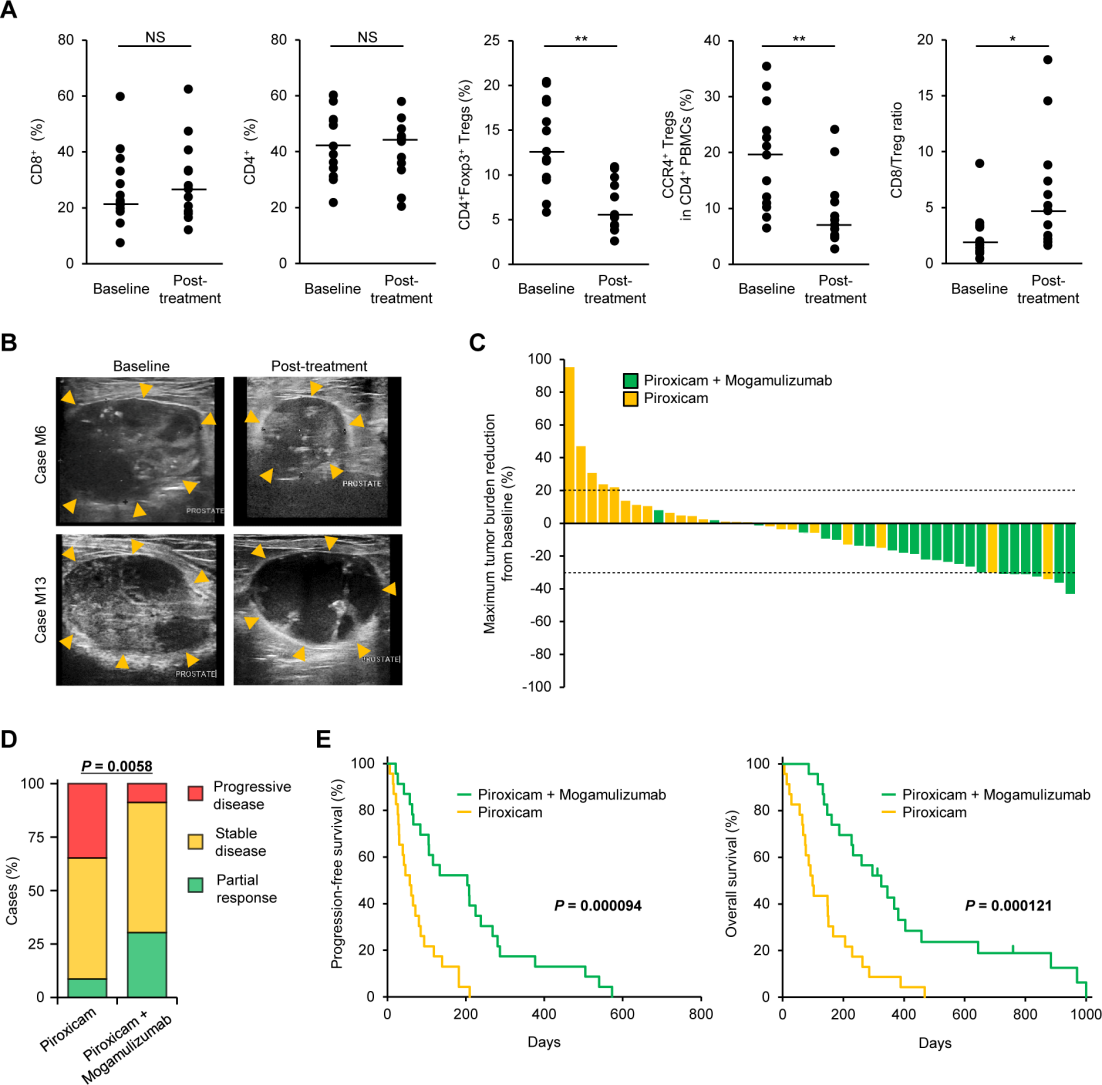
Anti-CCR4 therapy induces clinical responses and improves survival in dogs with prostate cancer. (A) Circulating CD8^+^ cells, CD4^+^ cells, CD4^+^Foxp3^+^ Tregs, CCR4^+^ Tregs, and CD8:Treg ratio at baseline and after mogamulizumab administration. (B) Representative ultrasonographic images of prostate masses (arrowheads) in dogs treated with mogamulizumab and piroxicam. In case M6, the prostate mass shrunk after four cycles of treatment compared with baseline. In case M13, the prostate size did not change, but necrosis was observed inside the mass after four cycles of treatment. (C) Waterfall plot showing the maximum percentage of tumor burden reduction from baseline in dogs treated with piroxicam (n=23, yellow) or in dogs treated with mogamulizumab and piroxicam (n=23, green). Dashed lines indicate –30% (partial response) and +20% (progressive disease). (D) Clinical responses in dogs treated with piroxicam (n=23) or mogamulizumab and piroxicam (n=23). Cochran-Armitage test. (E) Progression-free survival (left) and overall survival (right) in dogs treated with piroxicam (n=23, yellow) or mogamulizumab and piroxicam (n=23, green). Log-rank test. NS, *P<0.05, **P<0.01; non-parametric Mann-Whitney U test. NS, not significant; Treg, regulatory T cell.

A humanized anti-human CCR4, mogamulizumab, is commercially available for the treatment of CCR4^+^ adult T-cell leukemia/lymphoma.^6^ We previously confirmed that mogamulizumab crossreacts with canine CCR4 and depletes Tregs in dogs.^21^ To assess the clinical efficacy of the anti-CCR4 treatment in canine prostate cancer, we compared 23 dogs that received mogamulizumab and piroxicam with the control arm of 23 age-matched, sex-matched, and tumor stage-matched dogs that received piroxicam alone (online supplemental table S4). Mogamulizumab reduced circulating CD4^+^Foxp3^+^ Tregs and CCR4^+^ Tregs but not CD8^+^ cytotoxic T cells and CD4^+^ helper T cells in dogs with prostate cancer (figure 3A). CD8:Treg ratio was increased after mogamulizumab treatment (figure 3A). In two cases of tumor tissue collected before and after treatment, we confirmed that mogamulizumab reduced the number of Foxp3^+^ and CCR4^+^ cells (online supplemental figure S5). Typically, dogs treated with mogamulizumab in combination with piroxicam had a reduction in the tumor burden (figure 3B, upper, and online supplemental movie S1). Three dogs (case ID. M3, M13, and M15) exhibited fluid retention in the prostate due to necrosis of the tumor, although there was no reduction in the mass (figure 3B, lower, and online supplemental movie S2). Compared with dogs treated with piroxicam alone, those administered mogamulizumab/piroxicam treatment had a greater reduction in the size of the primary tumor (figure 3C). The median percentage of maximum tumor reduction in dogs treated with mogamulizumab/piroxicam and in those treated with piroxicam alone was –22.7% (range –43.6% to 8.5%) and 2.9% (range –34.7% to 95.9%), respectively. In 23 dogs with mogamulizumab/piroxicam, 7 (30%) obtained PR; 14 (61%) had SD; and 2 (9%) had PD. In 23 dogs with piroxicam alone, 2 (9%) obtained PR; 13 (56%) had SD; and 8 (35%) had PD. The clinical response to mogamulizumab/piroxicam was higher than the response to piroxicam alone (figure 3D). At the end of the study (March 1, 2021), 3 (13%) dogs treated with mogamulizumab were alive. The median PFS in dogs that received mogamulizumab/piroxicam and in those administered piroxicam alone was 204 (range 21–573) days and 57 (range 6–210) days, respectively. The median OS in dogs that received mogamulizumab/piroxicam and in those treated with piroxicam alone was 312 (range 86–1000) days and 99 (range,6–468) days, respectively. The PFS and OS in dogs treated with mogamulizumab/piroxicam were longer than in those treated with piroxicam alone (figure 3E). Multivariate analyses, including the candidate covariates (age, castration, BRAF^V595E^ mutation, tumor burden, and mogamulizumab treatment), showed that mogamulizumab treatment was individually associated with prolonged PFS (HR 0.27, 95% CI 0.13 to 0.57; p=0.0005) and OS (HR 0.28, 95% CI 0.14 to 0.55; p=0.0002).

10.1136/jitc-2021-003731.supp7 Supplementary video

10.1136/jitc-2021-003731.supp8 Supplementary video

Among 23 dogs treated with mogamulizumab/piroxicam, 17 (74%) had an adverse event (table 1). All treatment-related adverse events were grade 1 or 2. The most frequent adverse events were increased alkaline phosphatase (35%), increased alanine transaminase (17%), vomiting (17%), and anorexia (13%). As suspected immune-related adverse events, pancreatitis (grade 2), urticaria (grade 2), and rash (grade 1), and infusion reaction (grade 1) were observed in one case each (4%). Because mogamulizumab is a humanized antibody, the risk of allergic reactions in dogs was assumed; however, no serious allergic reactions, such as anaphylaxis, were observed. There were no treatment-related grade 3–5 adverse events, and no dogs experienced events that led to discontinuation of treatment. Lymphopenia was observed in some cases (grade 1 in 17% and grade 2 in 13%), which was considered as a pharmacological effect of mogamulizumab.

**Table 1:** Adverse events in dogs treated with mogamulizumab (n=23)

	Number of cases (%)*			
Event	Grade 1	Grade 2	Grades 3–5	Total
Any event	11 (47.8)	9 (39.1)	0	17 (73.9)
Non-hematological				
Increased ALP	4 (17.4)	4 (17.4)	0	8 (34.8)
Increased ALT	2 (8.7)	2 (8.7)	0	4 (17.4)
Vomiting	2 (8.7)	2 (8.7)	0	4 (17.4)
Anorexia	1 (4.3)	2 (8.7)	0	3 (13.0)
Lethargy/fatigue	2 (8.7)	0	0	2 (8.7)
Pancreatitis	0	1 (4.3)	0	1 (4.3)
Urticaria	0	1 (4.3)	0	1 (4.3)
Rash	1 (4.3)	0	0	1 (4.3)
Diarrhea	1 (4.3)	0	0	1 (4.3)
Infusion reaction	1 (4.3)	0	0	1 (4.3)
Hematological				
Lymphopenia	4 (17.4)	3 (13.0)	0	7 (30.4)

*Toxicity grade based on published criteria.^35^

ALP, alkaline phosphatase; ALT, alanine transaminase.

Taken together, these results suggest that the anti-CCR4 treatment depletes Tregs, leads to clinical responses, and improves survival without severe adverse events in dogs with advanced prostate cancer.

### Urinary CCL17 and BRAF^V595E^ mutation are biomarkers for predicting outcome to the anti-CCR4 treatment

**Figure 4:**
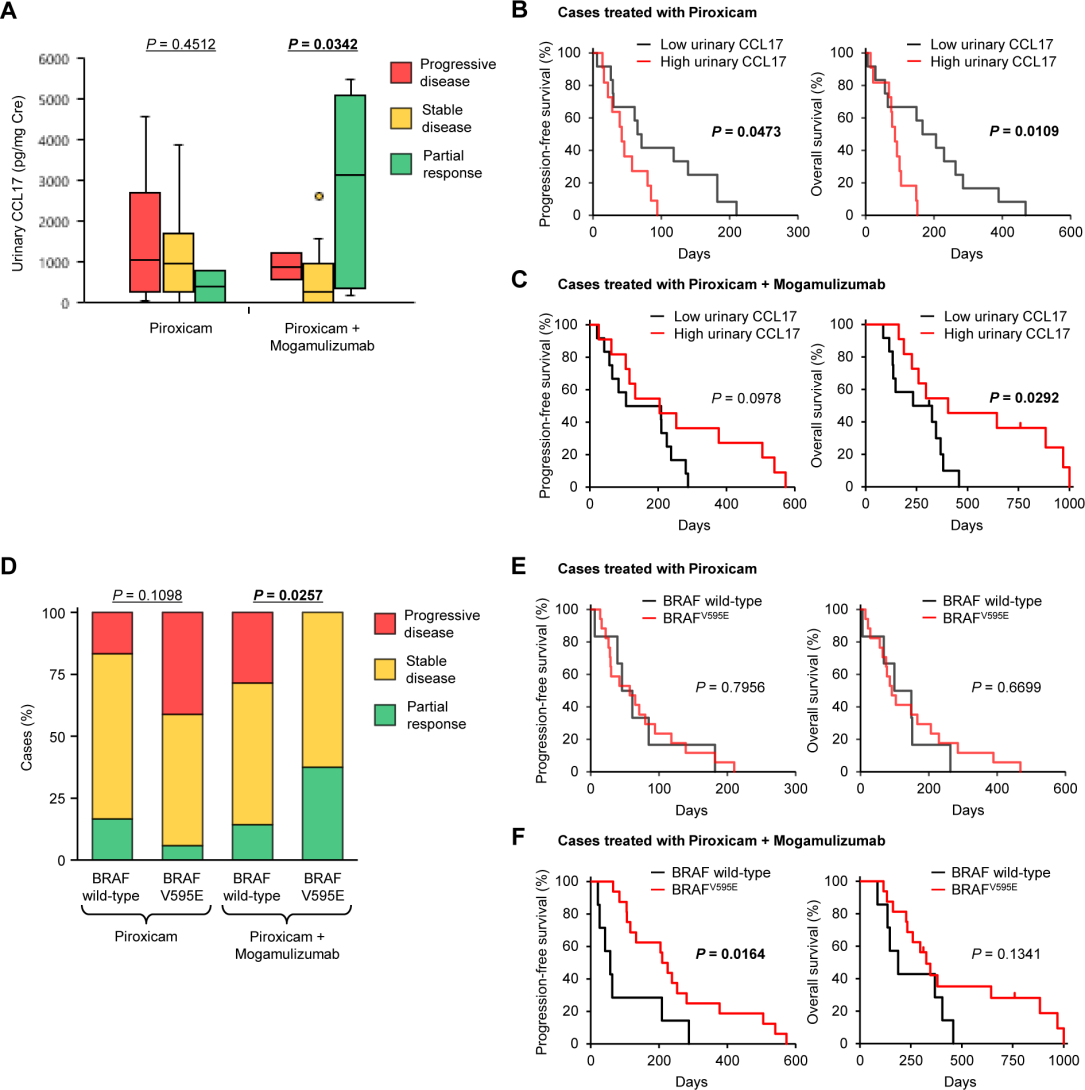
Urinary CCL17 concentrations and BRAF^V595E^ mutation are associated with clinical responses and outcomes of anti-CCR4 treatment. (A) Association of urinary CCL17 concentrations with response to treatment in dogs treated with piroxicam (n=23) or mogamulizumab and piroxicam (n=23), Kruskal-Wallis test. (B, C) Kaplan-Meier curves of PFS (left) and OS (right) according to urinary CCL17 concentrations in dogs treated with piroxicam (n=23) (B) or mogamulizumab and piroxicam (n=23) (C). Cases were classified as having a high (n=11) or low (n=12) concentration of urinary CCL17 according to the median, log-rank test. (D) Association of BRAF^V595E^ mutation with response to treatment in dogs treated with piroxicam (n=23) or mogamulizumab and piroxicam (n=23), Cochran-Armitage test. (E, F) Kaplan-Meier curves of PFS (left) and OS (right) according to BRAF gene status in dogs treated with piroxicam (n=23) (E) or mogamulizumab and piroxicam (n=23) (F). In dogs treated with piroxicam alone, 6 had wild-type BRAF and 17 had BRAF^V595E^ mutation. In dogs treated with mogamulizumab and piroxicam, 7 had wild-type BRAF and 16 had BRAF^V595E^ mutation. Log-rank test. OS, overall survival; PFS, progression-free survival

In a previous canine clinical trial of mogamulizumab for bladder cancer, urinary CCL17 was shown to be associated with clinical response.^21^ We investigated the association of pretreatment urinary CCL17 with the response in dogs with prostate cancer. No association was noted between urinary CCL17 and clinical response in dogs treated with piroxicam alone (figure 4A). In the cohort of mogamulizumab/piroxicam treatment, dogs with PR had more urinary CCL17 than did dogs with SD or PD (figure 4A). In dogs treated with piroxicam alone, PFS and OS for cases with high urinary CCL17 were shorter than those for cases with low urinary CCL17 (figure 4B). In contrast, high urinary CCL17 was associated with a longer OS in dogs treated with mogamulizumab/piroxicam (figure 4C).

We further examined the association between BRAF^V595E^ mutation and response. There was no association between BRAF^V595E^ mutation and clinical response in dogs treated with piroxicam alone, whereas BRAF^V595E^ mutation was associated with favorable response in dogs treated with mogamulizumab/piroxicam (figure 4D). Similarly, PFS and OS in dogs treated with piroxicam alone were not related to BRAF^V595E^ mutation (figure 4E). In dogs treated with mogamulizumab/piroxicam, PFS for cases with BRAF^V595E^ mutation was longer than that for cases with wild-type BRAF (figure 4F). These findings suggest that urinary CCL17 and BRAF^V595E^ mutation are useful biomarkers for predicting the clinical response and outcome to mogamulizumab treatment in dogs with prostate cancer.

### Human and canine prostate cancers exhibit common gene expression signatures

We hypothesized that transcriptional patterns of human and canine prostate cancers would be conserved. To systematically assess transcriptional patterns across species, we performed RNA-Seq analysis of canine prostate cancer and compared the data to a TCGA dataset of human prostate cancer.^28^ We selected statistically significant DEGs (q<0.01) between canine prostate cancer and normal tissues and extracted concordant genes in the expression data of humans. We identified 2297 genes. The expression patterns of these genes in canine and human prostate tissues were visualized with t-SNE (online supplemental figure S6). Gene expression patterns in normal canine and human prostate samples were clearly distinct, whereas prostate cancer samples were not clearly divided by species. These results suggest that similarities in gene expression signatures in a subset of prostate cancer might share biology across species.

### CCL17–CCR4 axis associates with tumor-infiltrating Tregs and poor prognosis in human prostate cancer

**Figure 5:**
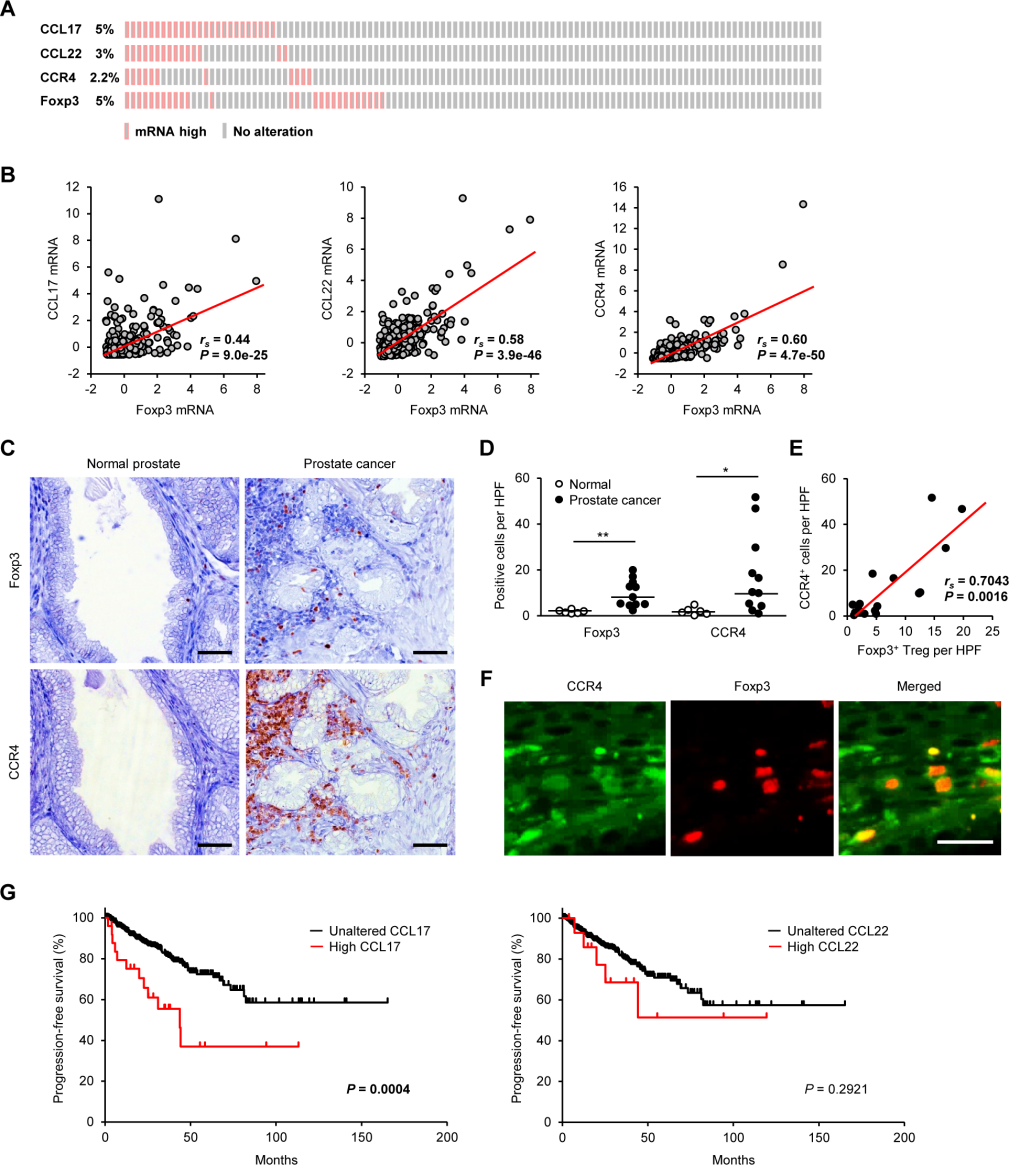
The CCL17–CCR4 axis is associated with tumor-infiltrating Tregs and prognosis in human patients with prostate cancer. (A) Expression data for CCL17, CCL22, CCR4, and Foxp3 mRNAs in human prostate cancer samples from the TCGA dataset (n=493). (B) Linear regression analysis between Foxp3 expression and CCR4-associated genes (CCL17, CCL22, and CCR4) in human prostate cancer samples from TCGA dataset (n=493); Spearman rank correlation coefficient. (C) Representative images of immunohistochemistry for Foxp3 and CCR4 in human prostate cancer. Scale bar, 50 µm. (D) The number of Foxp3^+^ Tregs and CCR4^+^ cells in the prostate of normal volunteers (n=6) and patients with prostate cancer (n=11). Median values are depicted by horizontal lines. (E) Correlation between Foxp3^+^ Tregs and CCR4^+^ cells in patients with prostate cancer. Spearman rank correlation coefficient. (F) Representative images of immunofluorescence for CCR4 (green) and Foxp3 (red) in human prostate cancer. Scale bar, 25 µm. (G) Kaplan-Meier curves of PFS according to the mRNA expression of CCL17 (left) and CCL22 (right) in human patients with prostate cancer from TCGA dataset (n=493). Log-rank test. *P<0.05, **P<0.01; non-parametric Mann-Whitney U test. PFS, progression-free survival; Treg, regulatory T cell.

Given the promising outcomes and favorable clinical responses of the anti-CCR4 treatment in the comparative canine trial, we examined whether the CCL17–CCR4 axis is associated with tumor-infiltrating Tregs and prognosis in patients with human prostate cancer. We searched for mRNA expression of CCL17, CCL22, CCR4, and Foxp3 in the publicly available transcriptomic dataset of human prostate cancer from TCGA PanCancer Atlas (n=493). High mRNA expression of CCL17, CCL22, CCR4, and Foxp3 was detected in 5.0%, 3.0%, 2.2%, and 5.0% of patients with prostate cancer, respectively (figure 5A). We found a correlation between mRNA expression of Foxp3 and CCL17 (*r*_s_=0.44, p=9.0×10^−25^), CCL22 (*r*_s_=0.58, p=3.9×10^−46^), and CCR4 (*r*_s_=0.60, p=4.7×10^−50^; figure 5B). Immunohistochemistry in serial sections of human tissues showed Foxp3^+^ and CCR4^+^ mononuclear lymphoid cells in prostate cancer but not in normal prostate (figure 5C). Compared with the normal prostate, Foxp3^+^ Tregs and CCR4^+^ cells were more frequent in patients with prostate cancer (figure 5D). The density of Foxp3^+^ Tregs was positively correlated with CCR4^+^ cells (figure 5E). Double-labeling immunofluorescence confirmed that Foxp3^+^ Tregs expressed CCR4 in the tumor microenvironment (figure 5F). To evaluate whether the ligands of CCR4 are associated with prognosis in human prostate cancer, we performed in silico survival analyses using TCGA dataset. We identified that high CCL17, but not CCL22, expression is associated with shorter PFS (figure 5G). Multivariate analysis, including the candidate covariates (age, tumor burden, lymph node metastasis, distant metastasis, and CCL17 expression), showed that the variables individually associated with a shortened PFS were tumor burden (HR 2.7, 95% CI 1.8 to 4.1; p<0.0001) and CCL17 expression (HR 2.2, 95% CI 1.2 to 4.1; p=0.013). These findings suggest that the CCL17–CCR4 axis is associated with tumor-infiltrating Tregs, and prognosis in patients with human prostate cancer and anti-CCR4 may have therapeutic value.

## Discussion

Immunotherapy is rapidly transforming cancer treatment across a range of tumor types. Prostate cancer tissues often contain immune cells, suggesting that this cancer is a target of host antitumor immunity.^39^ In 2010, sipuleucel-T, a cancer vaccine that targets prostatic acid phosphatase, was approved by Food and Drug Administration for the first immunotherapy of mCRPC.^40^ However, other immunotherapeutic approaches, including immune checkpoint inhibitors such as ipilimumab and pembrolizumab, have not been successful against mCRPC to date.^13–15^ This failure may be explained by highly immunosuppressive microenvironment in prostate cancer. An alternative approach is needed to evoke antitumor immunity and conquer the immunosuppressive microenvironment. In this study, we have shown that Foxp3^+^ Tregs infiltrate tumor tissues via the CCL17–CCR4 pathway, and anti-CCR4 treatment exerts an antitumor effect in dogs with advanced prostate cancer. In our canine clinical trial, objective response rates (ORRs) of mogamulizumab in combination with piroxicam were 30% (7 of 23 dogs), and the median PFS and OS were 204 days and 312 days, respectively. Although there is limited literature regarding treatment of canine prostate cancer, these results compare well with trials of piroxicam alone in this study (ORR, 9%; median PFS, 57 days; median OS, 99 days) or chemotherapy (carboplatin, mitoxantrone, or cyclophosphamide) in combination with COX inhibitors (ORR, 4%; median PFS, 89 days; median OS, 106 days).^41^ The reported median OS in dogs with prostate cancer following prostatectomy ranges from 19 to 231 days,^42–44^ indicating a relatively higher therapeutic efficacy of the mogamulizumab/piroxicam treatment. We also found that the CCL17–CCR4 pathway is associated with Treg infiltration and poor prognosis in human prostate cancer. Taken together, these findings suggest the potential of mogamulizumab for the treatment of prostate cancer. Ipilimumab and pembrolizumab inhibit Treg immunosuppression by blocking CTLA-4 and PD-1, respectively, but have no effect on other immune checkpoint molecules or immunosuppressive cytokines, such as IL-10 and TGF-β. In contrast, mogamulizumab may inhibit Treg immunosuppression more strongly by Treg depletion and suppression of intratumor infiltration.

A recent phase I study failed to show potent antitumor efficacy of mogamulizumab in combination with checkpoint inhibitors durvalumab or tremelimumab in patients with advanced solid tumors.^45^ However, there were only two patients with prostate cancer in the pilot trial, which is insufficient to conclude that mogamulizumab is not effective against human prostate cancer. A previous study has shown that checkpoint inhibitors upregulate the expression of CCL17 and CCL22 in tumors, leading to Treg migration into the microenvironment and resistance to treatment, and that blockade of CCR4 augmented the antitumor effects of checkpoint inhibitors.^9^ In this study, we showed that mogamulizumab is effective in dogs with prostate cancer, and gene expression signatures in some human patients with prostate cancer was similar to canines, indicating that patients with prostate cancer with canine-like subtypes might benefit from mogamulizumab treatment.

Mogamulizumab treatment in dogs was well tolerated, with all observed treatment-related adverse events being grade 1 or 2. Vomiting and anorexia were manageable with systemic antiemetic treatment alone. Immune-related adverse events, such as pancreatitis, urticaria, rash, and infusion reaction, were observed but were mild, and only one case was treated with diphenhydramine. In humans, immune-related adverse events, particularly skin disorders, have been associated with therapeutic responses to mogamulizumab treatment.^6^ In the present study, three dogs that developed immune-related adverse events (cases M3, M13, and M22) relatively had better PFS and OS (online supplemental table S4). A decrease in lymphocyte count, which may be a pharmacological effect of mogamulizumab, was observed in 30% of dogs; however, there was no increase in the risk of clinically evident infections. In humans, the most common adverse events associated with mogamulizumab treatment are infusion reactions (24%–89%), skin rashes (51%–63%), chills (59%), and fever (30%–82%), which are manageable and reversible.^6 46^ These safety data, including the lack of nephrotoxicity, suggest that patients with advanced prostate cancer who are older and more prone to renal failure may tolerate mogamulizumab treatment better than chemotherapy.

We show that the likelihood of CCR4 blockade therapy response (tumor burden reduction and survival) can be increased by determining the urinary CCL17 concentration or BRAF^V595E^ somatic mutation. These findings indicate a link between the BRAF^V595E^ mutation and Treg recruitment via the CCL17–CCR4 pathway in dogs with prostate cancer. The CCL17 concentration increased in urine but not in blood, suggesting that CCL17 produced locally in prostate cancer leaks into urine and is associated with the abundance of tumor-infiltrating Tregs. In humans, BRAF gene mutations occur in up to 8% of all cancers.^47^ Melanoma has the highest frequency of BRAF mutations (approximately 40%–60% of patients), with the majority harboring a BRAF^V600E^ mutation. Herein, we confirmed that BRAF gene alterations occur in up to 6.5% of human patients with prostate cancer. Given that more than 70% of canine prostate cancers harbor the BRAF^V595E^ mutation, the companion dog model could be a highly relevant platform for comparative cancer research to understand molecular events leading to BRAF mutation and further develop a BRAF-targeted therapy. It will be interesting to investigate whether BRAF inhibitors can enhance the antitumor effect of the CCR4 blockade therapy.

COX inhibitors, also known as non-steroidal anti-inflammatory drugs, are commonly used for cancer treatment, including prostate cancer, in veterinary medicine because of the antitumor effects, oral delivery, low cost, relatively low toxicity, and positive benefits on quality of life, making them more acceptable to pet owners (chemotherapy and surgery are often not accepted by pet owners). Approximately 90% of canine prostate cancers express COX-1 and COX-2 in tumor cells, and canine prostate cancer cases treated with COX inhibitors had an approximately 10-fold prolongation of OS compared with untreated cases.^48^ Proposed mechanisms of the antitumor effects of COX inhibitors include inhibition of tumor cell growth, induction of apoptosis, antiangiogenic effects, immunological effects, and modulation of cancer stem cells through suppressing the COX-2–PGE2 pathway. In this canine trial, therefore, cases treated with piroxicam, the standard treatment for canine prostate cancer, were used as the control group, and cases treated with piroxicam plus mogamulizumab were used as the test group. In humans, phase III randomized controlled trials evaluating the effect of daily aspirin on recurrence and survival after radical cancer therapy are ongoing in four tumor cohorts: gastro-oesophageal, colorectal, breast, and prostate cancers.^49^

Comparative oncology clinical trials in companion dogs play a growing role in cancer research and drug development efforts. In particular, an intact immune system and natural coevolution of tumor and microenvironment support exploration of novel immunotherapeutic strategies.^50^ Herein, we show that the CCL17–CCR4 axis is associated with Treg infiltration into the tumor tissues in both canine and human prostate cancers. Other canine malignancies, including non-Hodgkin’s lymphoma, glioma, bladder cancer, breast cancer, lung cancer, melanoma, and osteosarcoma, share genotypical and phenotypical similarities with human counterparts.^50^

In conclusion, we demonstrate that the CCL17–CCR4 pathway associates Treg infiltration into tumor tissues and adverse outcomes in canine and human prostate cancer. CCR4 blockade leads to clinical activity and improves survival without severe toxicity profiles in a canine model of advanced prostate cancer. These findings suggest that anti-CCR4 treatment may be a viable strategy for reducing immunosuppression caused by Tregs, thereby augmenting antitumor immunity in both dogs and humans.

## Data Availability

Data are available upon reasonable request. Availability of data and material: canine prostate cancer datasets used and/or analyzed during the current study are available from the corresponding author on reasonable request and will also be available at the DDBJ Sequenced Read Archive repository (https://www.ddbj.nig.ac.jp/index-e.html) with accession number DRA011773.
