# Supplementary material for: Anti-CCR4 treatment depletes regulatory T cells and leads to clinical activity in a canine model of advanced prostate cancer
Source: J Immunother Cancer. 2022 Jan 31;10(2):e003731. doi: 10.1136/jitc-2021-003731 (PMC8804701; doi:10.1136/jitc-2021-003731)
Supplement: Supplementary data [file jitc-2021-003731supp001.pdf]

Table S1. Clinical features of dogs with prostate cancer and analyzed factors performed in this study.

Case ID	Clinical features						Analyzed factors[†]					
	Age (years)	Sex*	Breed	Histology	$BRAF^{V595E}$ mutation	Treatment	IHC	Survival analysis	RNA-Seq	qPCR	Urine (ELISA)	Serum (ELISA)
PC1	11.2	MC	Wire Fox Terrier	Prostate cancer	Mutation	Radical cystoprostatectomy	+	+	+	+	+	+
PC2	11.1	MC	Yorkshire Terrier	Prostate cancer	Mutation	Radical cystoprostatectomy	+	+	+	+	+	+
PC3	12.7	MC	English Cocker Spaniel	Prostate cancer	Mutation	Radical cystoprostatectomy	+	+	+	+		
PC4	10.3	MC	Border Collie	Prostate cancer	Mutation	Radical cystoprostatectomy	+	+	+	+	+	+
PC5	11.8	MI	Border Collie	Prostate cancer	Wild-type	Radical cystoprostatectomy	+	+	+	+		
PC6	11.9	MC	Papillon	Prostate cancer	Mutation	Radical cystoprostatectomy	+	+	+	+	+	+
PC7	12.3	MI	Miniature Dachshund	Prostate cancer	Mutation	Radical cystoprostatectomy	+	+	+	+	+	+
PC8	11.4	MC	Labrador Retriever	Prostate cancer	Wild-type	Radical cystoprostatectomy	+	+	+	+	+	+
PC9	11.8	MC	Maltese	Prostate cancer	Wild-type	Radical cystoprostatectomy	+	+	+	+	+	+
PC10	10.8	MC	Miniature Dachshund	Prostate cancer	Mutation	Radical cystoprostatectomy	+	+	+	+		
PC11	15.9	MI	Miniature Dachshund	Prostate cancer	Wild-type	Radical cystoprostatectomy	+	+				
PC12	9.3	MC	Miniature Dachshund	Prostate cancer	Mutation	Radical cystoprostatectomy	+	+				
PC13	11.9	MC	Miniature Schnauzer	Prostate cancer	Mutation	Radical cystoprostatectomy	+	+				
PC14	10.4	MC	Pembroke Welsh Corgi	Prostate cancer	Mutation	Radical cystoprostatectomy	+	+				
PC15	12.5	MI	Miniature Schnauzer	Prostate cancer	Mutation	Radical cystoprostatectomy	+	+				
PC16	11.7	MC	Miniature Dachshund	Prostate cancer	Mutation	Radical cystoprostatectomy	+	+	+	+	+	+
PC17	9.0	MC	Pug	Prostate cancer	Mutation	Radical cystoprostatectomy	+	+	+	+	+	+
PC18	11.0	MC	Miniature Dachshund	Prostate cancer	Mutation	Radical cystoprostatectomy	+	+	+	+	+	+
PC19	9.9	MC	Miniature Dachshund	Prostate cancer	Mutation	Piroxicam			+	+	+	+
PC20	11.9	MC	Miniature Dachshund	Prostate cancer	Mutation	Firocoxib				+		
PC21	12.4	MC	Miniature Dachshund	Prostate cancer	Mutation	Piroxicam				+	+	+
PC22	11.4	MC	Cavalier King Charles Spaniel	Prostate cancer	Mutation	Piroxicam				+	+	+
PC23	8.2	MC	Jack Russell Terrier	Prostate cancer	Mutation	Piroxicam				+	+	+
PC24	12.6	MC	Brussels Griffon	Prostate cancer	Mutation	Piroxicam					+	+
PC25	12.0	MC	Papillon	Prostate cancer	Mutation	Piroxicam					+	+
PC26	13.3	MC	Chihuahua	Prostate cancer	Wild-type	Piroxicam					+	+
PC27	8.9	MI	Pembroke Welsh Corgi	Prostate cancer	Wild-type	Piroxicam					+	+
PC28	10.2	MC	Miniature Dachshund	Prostate cancer	Mutation	Piroxicam					+	+
N1	8.1	MI	Beagle	Normal	Wild-type	–	+		+	+	+	+
N2	10.2	MI	Beagle	Normal	Wild-type	–	+		+	+	+	+
N3	9.0	MI	Beagle	Normal	Wild-type	–	+		+	+	+	+
N4	9.1	MI	Beagle	Normal	Wild-type	–	+		+	+	+	+
N5	4.5	MI	Beagle	Normal	Wild-type	–	+			+	+	+
N6	4.5	MI	Beagle	Normal	Wild-type	–	+				+	+
N7	4.6	MI	Beagle	Normal	Wild-type	–	+				+	+
N8	4.6	MI	Beagle	Normal	Wild-type	–	+				+	+
N9	4.8	MI	Beagle	Normal	Wild-type	–	+				+	+
N10	8.8	MI	Beagle	Normal	Wild-type	–					+	+
N11	10.4	MC	Toy Poodle	Normal	Wild-type	–					+	
N12	8.9	MC	Maltese	Normal	Wild-type	–					+	
N13	10.1	MC	Miniature Dachshund	Normal	Wild-type	–					+	
N14	7.6	MC	Papillon	Normal	Wild-type	–					+	

*MC, male castrated; MI, male intact. [†]IHC, immunohistochemistry; RNA-Seq, RNA sequencing; qPCR, quantitative PCR; ELISA, enzyme-linked immunosorbent assay.

Maeda S, *et al. J Immunother Cancer* 2022; 10:e003731. doi: 10.1136/jitc-2021-003731
