# Supplementary material for: Anti-CCR4 treatment depletes regulatory T cells and leads to clinical activity in a canine model of advanced prostate cancer
Source: J Immunother Cancer. 2022 Jan 31;10(2):e003731. doi: 10.1136/jitc-2021-003731 (PMC8804701; doi:10.1136/jitc-2021-003731)
Supplement: Supplementary data [file jitc-2021-003731supp002.pdf]

Table S2. Characteristics of human patients with prostate cancer.

Case ID	Age (years)	Sex*	Pathological diagnosis	Gleason score	TNM classification	Minimum stage grouping	Foxp3⁺ cells (cells/HPF)†	CCR4⁺ cells (cells/HPF)†
hPC1	74	M	Adenocarcinoma of prostate	3+4=7/10	pT2apN0pMX	II	2.2	5.2
hPC2	60	M	Adenocarcinoma of prostate	3+4=7/10	pT2cpNXpMX	II	8	16.4
hPC3	52	M	Adenocarcinoma of prostate	3+4=7/10	pT2bpN0pMX	II	5	0.8
hPC4	59	M	Adenocarcinoma of prostate	3+4=7/10	pT2cpNXpMX	II	17	29.6
hPC5	61	M	Adenocarcinoma of prostate	3+4=7/10	pT3bpN0pMX	III	5.2	4.2
hPC6	75	M	Adenocarcinoma of prostate	3+4=7/10	pT2cpN0pMX	II	14.6	51.6
hPC7	54	M	Adenocarcinoma of prostate	3+4=7/10	pT2apN0pMX	II	4.4	18.4
hPC8	57	M	Adenocarcinoma of prostate	3+4=7/10	pT2bpN0pMX	II	19.8	46.6
hPC9	62	M	Adenocarcinoma of prostate	3+4=7/10	pT3bpNXpMX	III	4.8	2.2
hPC10	61	M	Adenocarcinoma of prostate	3+4=7/10	pT3bpNXpMX	III	12.4	9.8
hPC11	58	M	Adenocarcinoma of prostate	3+4=7/10	pT2cpN0pMX	II	12.6	10.2
hN1	62	M	Normal	–	–	–	3	0.8
hN2	54	M	Normal	–	–	–	1.2	0.2
hN3	65	M	Normal	–	–	–	1	4.8
hN4	66	M	Normal	–	–	–	1.4	1.4
hN5	70	M	Normal	–	–	–	2.2	2
hN6	65	M	Normal	–	–	–	2	2.6

*M, male. †HPF, high power field.

Maeda S, *et al. J Immunother Cancer* 2022; 10:e003731. doi: 10.1136/jitc-2021-003731
