# Supplementary material for: Anti-CCR4 treatment depletes regulatory T cells and leads to clinical activity in a canine model of advanced prostate cancer
Source: J Immunother Cancer. 2022 Jan 31;10(2):e003731. doi: 10.1136/jitc-2021-003731 (PMC8804701; doi:10.1136/jitc-2021-003731)
Supplement: Supplementary data [file jitc-2021-003731supp003.pdf]

Supplemental Methods

Control dogs

Fourteen dogs were used as healthy controls (Supplementary Table S1). These dogs were healthy, exhibited no clinical signs of gastrointestinal disease, such as diarrhoea, vomiting, anorexia or weight loss, and were not treated with any drugs. Routine urinalysis and blood examinations, including a complete blood count and measurements of blood urea nitrogen, creatinine, alanine aminotransferase and alkaline phosphatase levels, showed no abnormalities. These dogs were kept in multiples in cages that were large enough for them.

Immunohistochemistry

The expression of Foxp3 and CCR4 was examined by immunohistochemistry with 4 μm-thick paraffin-embedded sections.[1] Heat-induced antigen retrieval was performed by autoclaving the sections for 5 min at 121°C in 10 mM sodium citrate buffer (pH 6.0). Endogenous peroxidase activity was blocked by incubation with REAL Peroxidase-Blocking Solution (Dako) at room temperature for 10 min. The sections were blocked with 5% skim milk in Tris-buffered saline with 0.1% Tween 20 (TBST) at room temperature for 60 min and then incubated with primary antibodies, rat anti-Foxp3 (1:400 dilution, clone FJK-16s, eBioscience) or mouse anti-CCR4 (1:100 dilution, clone 1G1, BD Biosciences), at 4°C overnight. Secondary antibodies were applied as follows: EnVision polymer reagent for mouse (Dako) at room temperature for 45 min or a biotin-labeled anti-rat IgG (Vector Laboratories) at 37°C for 30 min followed by HRP-labeled streptavidin (Dako) at room temperature for 30 min. The reaction products were visualized with 3,3′-diaminobenzidine (DAB). For double immunofluorescence, antigen

retrieval, endogenous peroxidase blocking, and milk blocking were performed as described above. Primary antibodies, rat anti-Foxp3 (1:100 dilution, clone FJK-16s, eBioscience) and mouse anti-CCR4 (1:100 dilution, clone 1G1, BD Biosciences), were applied at 4°C overnight. Immunofluorescence was performed using secondary antibodies, Alexa Fluor 594 goat anti-rat IgG (1:500, Invitrogen) and Alexa Fluor 488 donkey anti-mouse IgG (1:500, Invitrogen). Images were captured using a fluorescence microscope (BZ-X800; Keyence).

For human prostate tissues, antigen retrieval, endogenous peroxidase blocking, and milk blocking were performed as described above. As primary antibodies, rabbit anti-human Foxp3 (1:100 dilution, clone 1054C, R&D Systems) or mouse anti-human CCR4 (1:100 dilution, clone 1G1, BD Biosciences), was applied at 4°C overnight. Immunohistochemistry was performed by using secondary antibodies, EnVision polymer reagent for rabbit or mouse (Dako) at room temperature for 45 min, followed by DAB detection. Immunofluorescence was performed using secondary antibodies, Alexa Fluor 598 goat anti-rabbit IgG (1:500, Invitrogen) and Alexa Fluor 488 donkey anti-mouse IgG (1:500, Invitrogen).

Cells with clear lymphocyte morphology, distinct nuclear staining for Foxp3 or cytoplasmic staining for CCR4 were evaluated as positive. Foxp3$^+$ or CCR4$^+$ cells were quantified in 10 representative fields of each slide (40× magnification) using the ImageJ software.[2]

Bioinformatic analysis of human prostate cancer

Datasets for human metastatic or nonmetastatic prostate cancer were accessed and BRAF gene alterations were analyzed through cBioPortal.[3-10] A normalized mRNA expression dataset for

human prostate cancer (TCGA, PanCancer Atlas) was accessed and downloaded from the cBioPortal and used to evaluate associations between CCL17, CCL22, CCR4, and Foxp3 expression. This data set includes mRNA profiles for 493 patients with prostate cancer.[6] PFS analysis was done for CCL17 and CCL22 transcripts for the cases. Detailed information of prostate cancer patients including pathology diagnosis, clinical stage, and survival data can be downloaded from the cBioPortal website (https://www.cbioportal.org/).

Comparative genomic analysis between dogs and humans

RNA-seq count data for human prostate samples were obtained from GDC data portal (https://portal.gdc.cancer.gov/). Five hundred fifty count data for HTseq analyzed (498 prostate cancer and 52 normal prostate tissues) from TCGA-PRAD project were downloaded for this study.[11] These data were also analyzed in the same way as canine data.

The most statistically significant differentially expressed genes (DEGs) between canine prostate cancer and normal tissues ($q < 0.01$) were extracted. These canine genes were converted to HGNC symbols using BioMart[12] and then the expression data of concordant genes between dogs and humans were extracted. This analysis resulted in 2,297 gene symbols, which were then used for t-distributed stochastic neighbor embedding (t-SNE) analysis (Rtsne package; ver. 0.15) of all samples.[13]

Flow cytometry

For Foxp3 intracellular staining, fixation and permeabilization of canine peripheral blood mononuclear cells (PBMCs) were with the Fixation/Permeabilization Solution Kit (BD Biosciences). We used the following monoclonal antibodies (mAbs) for flow cytometry of

Maeda S, *et al. J Immunother Cancer* 2022; 10:e003731. doi: 10.1136/jitc-2021-003731

PBMCs: CD4 (clone CA13.1E4; Leukocyte Antigen Biology Laboratory), CD8 (clone CA9.JD3; Leukocyte Antigen Biology Laboratory), CCR4 (clone 1G1; BD Biosciences), and Foxp3 (clone FJK-16s; eBioscience). All analyses were performed with FACSVerse (BD Biosciences).

References

1. Maeda, S., Murakami, K., Inoue, A., Yonezawa, T.Matsuki, N. Ccr4 blockade depletes regulatory t cells and prolongs survival in a canine model of bladder cancer. *Cancer Immunol Res* 2019;7:1175-87.
2. Schneider, C.A., Rasband, W.S.Eliceiri, K.W. Nih image to imagej: 25 years of image analysis. *Nat Methods* 2012;9:671-5.
3. Abida, W., Armenia, J., Gopalan, A.*, et al.* Prospective genomic profiling of prostate cancer across disease states reveals germline and somatic alterations that may affect clinical decision making. *JCO Precis Oncol* 2017;2017:
4. Abida, W., Cyrta, J., Heller, G.*, et al.* Genomic correlates of clinical outcome in advanced prostate cancer. *Proc Natl Acad Sci U S A* 2019;116:11428-36.
5. Armenia, J., Wankowicz, S.A.M., Liu, D.*, et al.* The long tail of oncogenic drivers in prostate cancer. *Nat Genet* 2018;50:645-51.
6. Consortium, I.T.P.-C.A.o.W.G. Pan-cancer analysis of whole genomes. *Nature* 2020;578:82-93.
7. Grasso, C.S., Wu, Y.M., Robinson, D.R.*, et al.* The mutational landscape of lethal castration-resistant prostate cancer. *Nature* 2012;487:239-43.

8. Nguyen, B., Mota, J.M., Nandakumar, S., *et al.* Pan-cancer analysis of cdk12 alterations identifies a subset of prostate cancers with distinct genomic and clinical characteristics. *Eur Urol* 2020;78:671-9.
9. Robinson, D., Van Allen, E.M., Wu, Y.M., *et al.* Integrative clinical genomics of advanced prostate cancer. *Cell* 2015;161:1215-28.
10. Stopsack, K.H., Nandakumar, S., Wibmer, A.G., *et al.* Oncogenic genomic alterations, clinical phenotypes, and outcomes in metastatic castration-sensitive prostate cancer. *Clin Cancer Res* 2020;26:3230-8.
11. Anders, S., Pyl, P.T.Huber, W. Htseq--a python framework to work with high-throughput sequencing data. *Bioinformatics* 2015;31:166-9.
12. Smedley, D., Haider, S., Durinck, S., *et al.* The biomart community portal: An innovative alternative to large, centralized data repositories. *Nucleic Acids Res* 2015;43:W589-98.
13. Maaten, L. Accelerating t-sne using tree-based algorithms. *J Mach Learn Res* 2014;15:3221-45.

Maeda S, *et al. J Immunother Cancer* 2022; 10:e003731. doi: 10.1136/jitc-2021-003731
