# Supplementary material for: Anti-CCR4 treatment depletes regulatory T cells and leads to clinical activity in a canine model of advanced prostate cancer
Source: J Immunother Cancer. 2022 Jan 31;10(2):e003731. doi: 10.1136/jitc-2021-003731 (PMC8804701; doi:10.1136/jitc-2021-003731)
Supplement: Supplementary data [file jitc-2021-003731supp004.pdf]

Table S3. Primer pair sequences used for RT-qPCR.

Primer set	GenBank accession number		Primer sequence (5′–3′)
IL-10	NM_001003077	Forward	CGA CCC AGA CAT CAA GAA CC
		Reverse	CAC AGG GAA GAA ATC GGT GA
TGF-β	NM_001003309	Forward	CTG GAG TCG TGA GGC AGT G
		Reverse	GCA GTG TGT TAT CTT TGC TGT CA
CCL3	NM_001005251	Forward	CAA GCA GAT TCC ACG CAA GGT
		Reverse	TAA TAC CGG GCT TGG AGC AT
CCL4	NM_001005250	Forward	CGT CCT TTC TCT CCT TGT GC
		Reverse	GAA TCT TCC GCA GGG TGT AA
CCL5	NM_001003010	Forward	GGT CTC CGC AGC TAC CTT T
		Reverse	AAA GCA GCA GGG TGT GGT
CCL7	NM_001010960	Forward	CCC ATC CAG AAG CTG AAG AG
		Reverse	CGT CCT TAG CCA GTT TGG TC
CCL8	NM_001005255	Forward	GTC CTT GCT CAG CCA GAT TC
		Reverse	ACT GGC TGT TGG TGA TCC TC
CCL13	NM_001003966	Forward	GCC CTA TTC ACT TGC TGC TT
		Reverse	AAT CCT GGA CCC ATT TCT CC
CCL14	XM_537723	Forward	TCA CGA GGA CCT TAC CAT CC
		Reverse	GGC CAT TTT TGG TGA TGA AG
CCL17	NM_001003051	Forward	GGC TGA CAA GGT GGT ACA AGA CTT C
		Reverse	CAG ATG GAC TTG CCT TGG ACA G
CCL22	XM_003433778	Forward	TAT GGT GCC AAC GTG GAA GA
		Reverse	GAT CTC CCG ATC CTT GAC AGT TAG
CCL24	NM_001003967	Forward	CCT GCT GCA TGT TCT TCA TTT C
		Reverse	TTC TGG TTC TTC TTG GTG GTG A
CCL28	NM_001005257	Forward	CAG ACA GGA CTC ACT CTC GCT CTC
		Reverse	TGT GAA ACC TCA GTG CAA CAG CTA
CXCL8	NM_001003200	Forward	CTT CCA AGC TGG CTG TTG CTC
		Reverse	TGG GCC ACT GTC AAT CAC TCT C
CXCL10	NM_001010949	Forward	ATT GAG ATG ATT CCT GCA AGT
		Reverse	TCA GAC ATC TTT TCT CCC CAC TC
CXCL13	XM_845089	Forward	GGG TGC CCA AAA AGA GAA ATC
		Reverse	GAT GGG AGG GTT CAA GCA TAC A
CXCL16	XM_014113226	Forward	GAG AGC CAG AAG CAG CAG AT
		Reverse	GTG ACT GCT CCC TCC TCT TG
CX3CL1	AB648939	Forward	CTT CCT TGG CCT CCT CTT CT
		Reverse	GGC ACC AGG ACA TAC GAG TT
RPL13A	AJ388525	Forward	GCC GGA AGG TTG TAG TCG T
		Reverse	GGA GGA AGG CCA GGT AAT TC
RPL32	XM_848016	Forward	TGG TTA CAG GAG CAA CAA GAA A
		Reverse	GCA CAT CAG CAG CAC TTC A

IL, interleukin; RPL, ribosomal protein L.
