# Supplementary material for: Anti-CCR4 treatment depletes regulatory T cells and leads to clinical activity in a canine model of advanced prostate cancer
Source: J Immunother Cancer. 2022 Jan 31;10(2):e003731. doi: 10.1136/jitc-2021-003731 (PMC8804701; doi:10.1136/jitc-2021-003731)
Supplement: Supplementary data [file jitc-2021-003731supp005.pdf]

Table S4. Characteristics of dogs with prostate cancer in the clinical trial.

Case ID	Age (years)	Sex*	Breed	TNM classification (at first visit)	BRAF^{V595E} mutation	Treatment	Response†	Progression-free survival (days)	Overall survival (days)
M1	9.8	MC	Papillon	T2N1M0	Mutation	Mogamulizumab Piroxicam	PR	105	296
M2	11.5	MC	Miniature Dachshund	T4N0M0	Mutation	Mogamulizumab Piroxicam	SD	66	381
M3	7.2	MC	Miniature Dachshund	T3N0M0	Mutation	Mogamulizumab Piroxicam	SD	505	1000
M4	12.1	MC	Miniature Dachshund	T4N0M0	Mutation	Mogamulizumab Piroxicam	PR	116	162
M5	13	MC	Miniature Dachshund	T3N0M0	Wild-type	Mogamulizumab Piroxicam	SD	287	368
M6	9.6	MI	Miniature Dachshund	T3N1M0	Mutation	Mogamulizumab Piroxicam	PR	209	232
M7	12.9	MC	Chihuahua	T4N1M0	Wild-type	Mogamulizumab Piroxicam	SD	57	147
M8	14.8	MC	Miniature Schnauzer	T4N0M0	Wild-type	Mogamulizumab Piroxicam	SD	208	458
M9	12	MC	Papillon	T4N1M1	Wild-type	Mogamulizumab Piroxicam	SD	42	86
M10	11.6	MC	Chihuahua	T3N1M1	Mutation	Mogamulizumab Piroxicam	PR	133	227
M11	13.7	MC	Toy Poodle	T4N1M0	Wild-type	Mogamulizumab Piroxicam	PR	63	404
M12	12.6	MC	Miniature Schnauzer	T2N0M0	Mutation	Mogamulizumab Piroxicam	SD	106	116
M13	12.2	MC	Miniature Dachshund	T3N0M0	Mutation	Mogamulizumab Piroxicam	SD	540	644
M14	14.8	MC	Miniature Dachshund	T2N0M0	Mutation	Mogamulizumab Piroxicam	SD	204	884
M15	13.4	MC	Miniature Dachshund	T4N1M1	Mutation	Mogamulizumab Piroxicam	SD	238	345
M16	12.4	MI	Miniature Dachshund	T4N1M1	Wild-type	Mogamulizumab Piroxicam	PD	26	187
M17	9.8	MC	Miniature Dachshund	T4N1M0	Mutation	Mogamulizumab Piroxicam	SD	225	325
M18	13.5	MC	Toy Poodle	T4N0M0	Mutation	Mogamulizumab Piroxicam	SD	573	>759
M19	13.6	MC	Miniature Schnauzer	T4N1M1	Mutation	Mogamulizumab Piroxicam	PR	84	133
M20	13.5	MI	Pug	T4N1M0	Wild-type	Mogamulizumab Piroxicam	PD	21	136
M21	13.8	MC	Chihuahua	T2N0M0	Mutation	Mogamulizumab Piroxicam	SD	253	260
M22	13.3	MC	Pomeranian	T1N0M0	Mutation	Mogamulizumab Piroxicam	PR	377	970
M23	13.2	MC	Toy Poodle	T2N0M0	Mutation	Mogamulizumam Piroxicam	SD	281	>312
P1	8.5	MC	Pembroke Welsh Corgi	T3N1M0	Mutation	Piroxicam	PD	22	22
P2	11.1	MI	French Bulldog	T4N0M0	Mutation	Piroxicam	SD	71	166
P3	9.8	MC	Miniature Dachshund	T4N1M1	Mutation	Piroxicam	PD	14	14
P4	11.6	MC	Miniature Dachshund	T3N1M0	Wild-type	Piroxicam	SD	46	68

P5	11.8	MC	Miniature Dachshund	T3N0M1	Mutation	Piroxicam	SD	65	65
P6	12.1	MC	Miniature Dachshund	T2N1M0	Mutation	Piroxicam	PD	30	206
P7	11.4	MC	Cavalier King Charles Spaniel	T4N1M1	Wild-type	Piroxicam	PD	6	6
P8	11.4	MC	Wire Fox Terrier	T4N0M0	Mutation	Piroxicam	SD	118	285
P9	11.5	MC	Yorkshire Terrier	T4N0M0	Wild-type	Piroxicam	SD	61	149
P10	12.4	MC	English Cocker Spaniel	T4N0M0	Mutation	Piroxicam	SD	94	148
P11	12.1	MI	Border Collie	T4N0M0	Wild-type	Piroxicam	SD	39	151
P12	11.8	MC	Papillon	T4N0M0	Mutation	Piroxicam	SD	57	92
P13	13.8	MC	Beagle	T2N0M0	Wild-type	Piroxicam	PR	182	263
P14	12.3	MC	Miniature Dachshund	T4N1M0	Mutation	Piroxicam	PD	29	29
P15	12.5	MC	Brussels Griffon	T4N1M0	Mutation	Piroxicam	PD	28	75
P16	8.1	MC	Jack Russel Terrier	T4N1M0	Mutation	Piroxicam	PD	16	103
P17	8.6	MC	Miniature Dachshund	T2N1M0	Mutation	Piroxicam	PD	26	56
P18	12.2	MC	Miniature Dachshund	T1N0M0	Mutation	Piroxicam	SD	182	229
P19	12.4	MI	Miniature Schnauzer	T3N1M1	Mutation	Piroxicam	SD	42	77
P20	15.7	MC	Miniature Dachshund	T2N0M0	Mutation	Piroxicam	SD	210	389
P21	8.9	MI	Pembroke Welsh Corgi	T4N1M1	Wild-type	Piroxicam	SD	85	99
P22	13	MC	Toy Poodle	T4N0M0	Mutation	Piroxicam	PR	139	468
P23	12.2	MC	Miniature Dachshund	T2N0M0	Mutation	Piroxicam	SD	80	86

*MC, male castrated; MI, male intact. †SD, stable disease; PR, partial response; PD, progressive disease.
