# Supplementary material for: Anti-CCR4 treatment depletes regulatory T cells and leads to clinical activity in a canine model of advanced prostate cancer
Source: J Immunother Cancer. 2022 Jan 31;10(2):e003731. doi: 10.1136/jitc-2021-003731 (PMC8804701; doi:10.1136/jitc-2021-003731)
Supplement: Supplementary data [file jitc-2021-003731supp006.pdf]

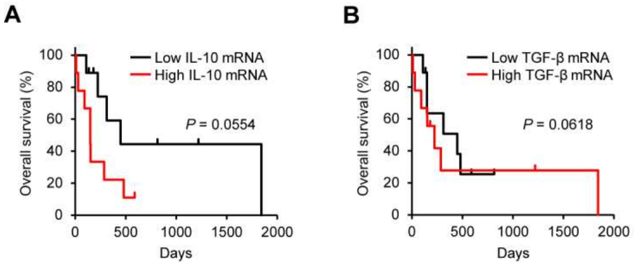

Figure S1. The mRNA expression of IL-10 and TGF-β is not associated with overall survival. (**A** and **B**) Kaplan–Meier curves of overall survival according to the mRNA expression of IL-10 (**A**) and TGF-β (**B**) in dogs with prostate cancer (*n* = 18). Cases were classified as having a high or low mRNA expression according to the median number (*n* = 9 each). Log-rank test.

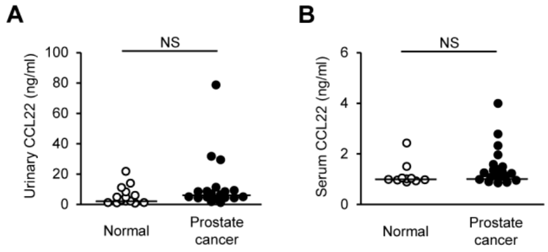

Figure S2. Urinary and serum CCL22 concentration in dogs with prostate cancer.

(**A**) Urinary CCL22 concentration in normal dogs (n = 14) and in dogs with prostate cancer (n = 19). Mean values are depicted by horizontal lines. NS, not significant, nonparametric Mann–Whitney *U* test. (**B**) Serum CCL22 concentration in normal dogs (n = 10) and in dogs with prostate cancer (n = 19). NS, not significant, nonparametric Mann–Whitney *U* test.

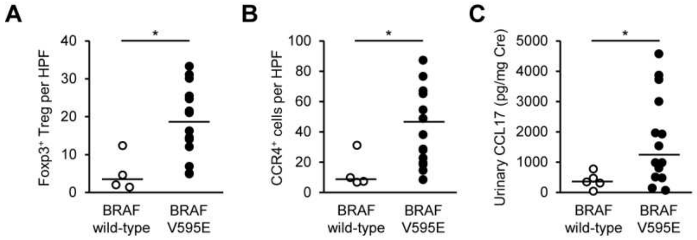

Figure S3. BRAF^{V595E} mutation is associated with intratumoral Foxp3$^+$ Tregs, CCR4$^+$ cells, and urinary CCL17 concentration in dogs with prostate cancer. (**A**–**C**) The number of intratumoral Foxp3$^+$ Tregs (**A**), CCR4$^+$ cells (**B**), and urinary CCL17 concentrations (**C**) in canine prostate cancer cases with wild-type BRAF and BRAF^{V595E} mutation. *P < 0.05, nonparametric Mann–Whitney U test.

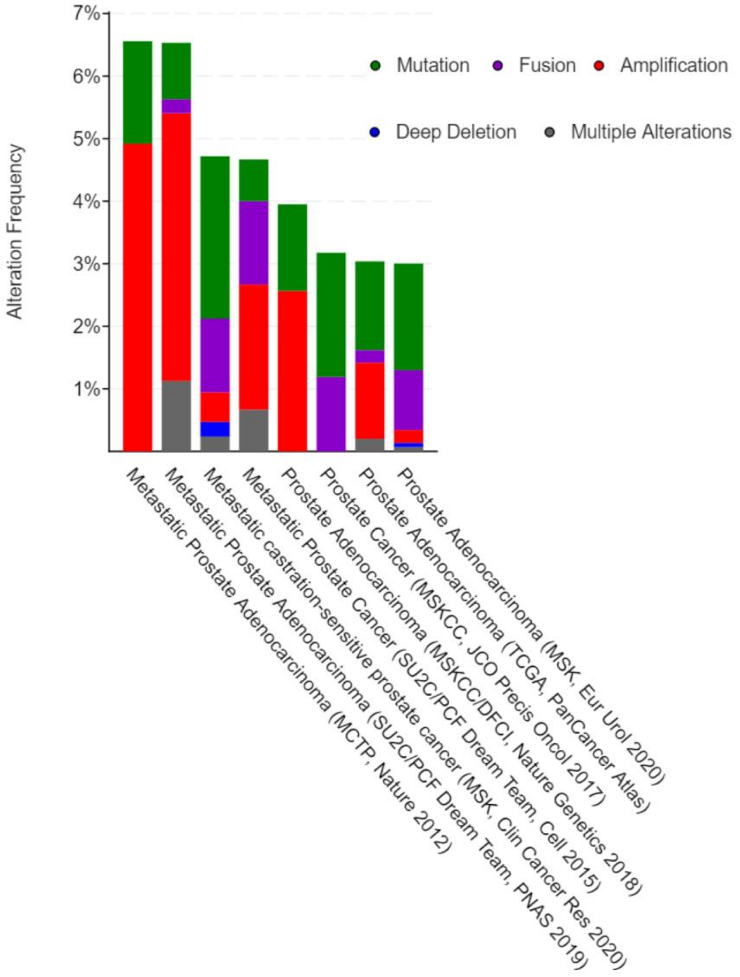

Figure S4. BRAF gene alterations in human prostate cancer. Histogram displaying frequency of BRAF gene somatic mutation, fusion, copy-number amplification, deletion, and multiple alterations across eight human prostate cancer genomic datasets.

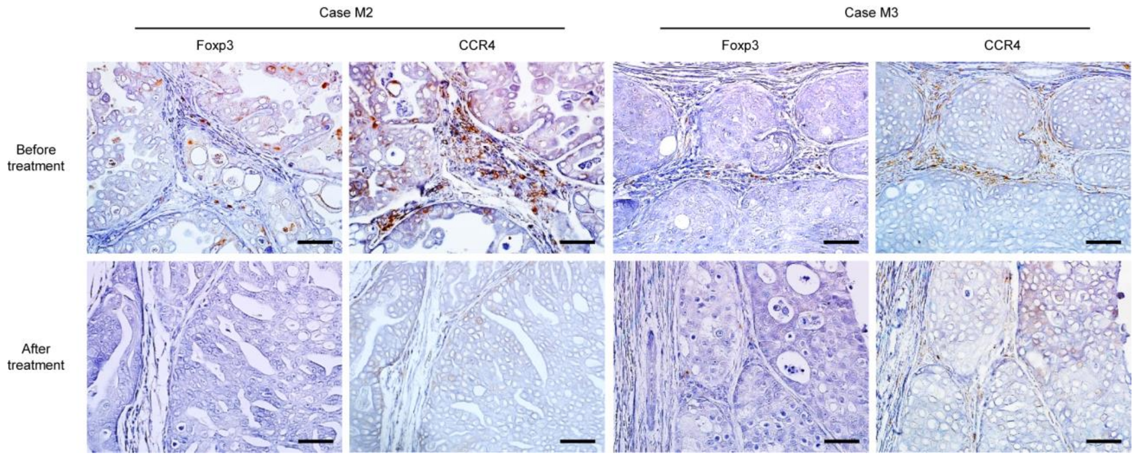

Figure S5. Intratumoral Foxp3$^+$ and CCR4$^+$ cells in two dogs with prostate cancer before and after mogamulizumab treatment. Before treatment, a number of Foxp3$^+$ and CCR4$^+$ cells were observed in the tumor tissue. After three (Case M2) and 33 cycles (Case M3) of mogamulizumab treatment, both Foxp3$^+$ and CCR4$^+$ cells were clearly decreased when compared with the pre-treatment tissue.

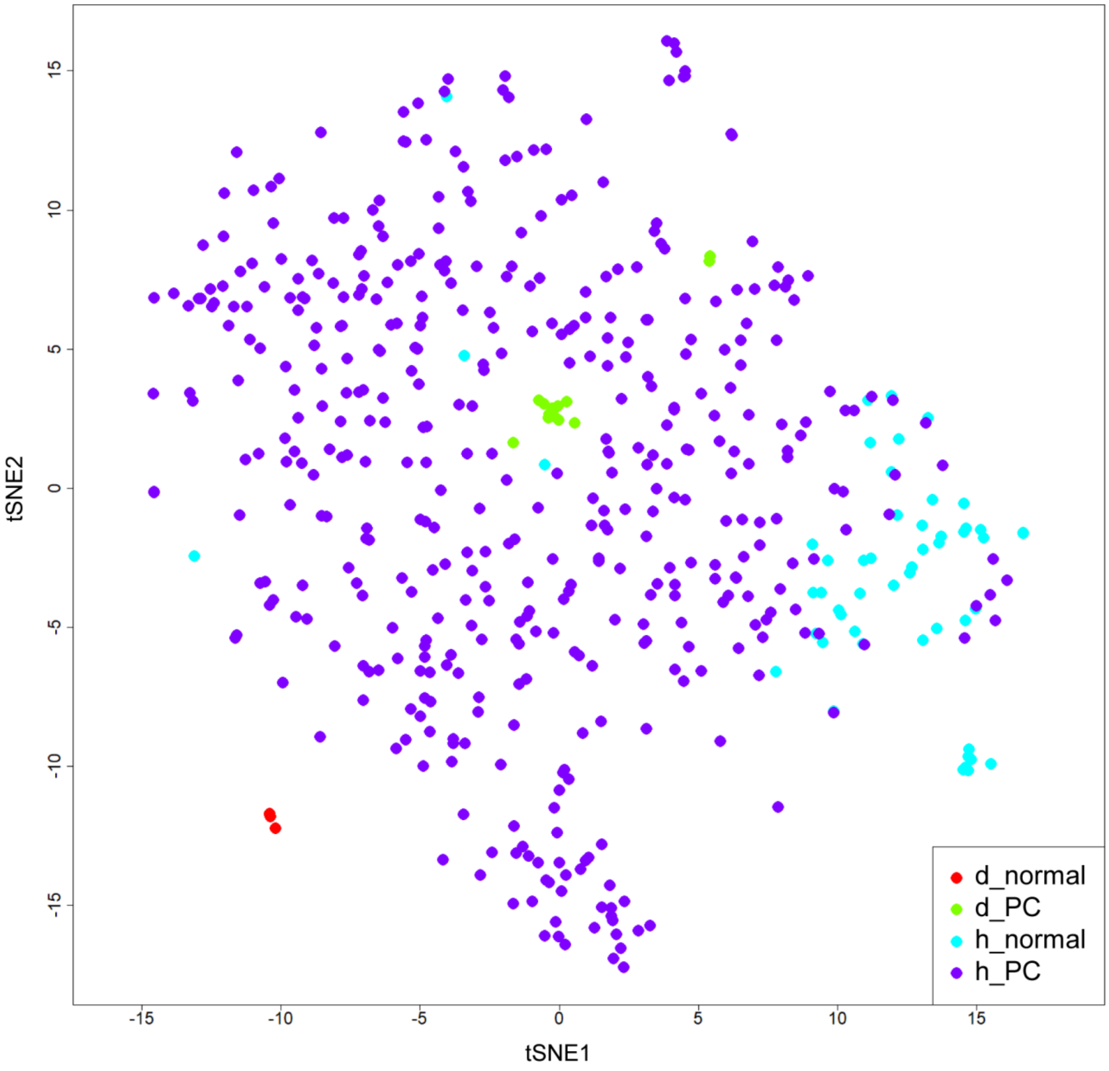

Figure S6. Cross-species t-SNE analysis of canine and human prostate tissues. Differentially expressed genes (q < 0.01) between canine prostate cancer and normal prostate were selected and extracted concordant genes in the expression data of humans. The expression patterns of 2,297 differentially expressed genes in the cross-species tissues were visualized with t-SNE. Color-coded sphere showed each type of prostate tissue as following; red: canine normal prostate, green: canine prostate cancer, blue: human normal prostate, purple: human prostate cancer.

Maeda S, *et al. J Immunother Cancer* 2022; 10:e003731. doi: 10.1136/jitc-2021-003731
